# Pharmacist care and the management of coronary heart disease: a systematic review of randomized controlled trials

**DOI:** 10.1186/1472-6963-13-461

**Published:** 2013-11-04

**Authors:** Hongwen Cai, Haibin Dai, Yangmin Hu, Xiaofeng Yan, Huimin Xu

**Affiliations:** 1 Department of Cardiovascular Medicine, First Affiliated Hospital, Zhejiang Chinese Medical University, Hangzhou, Zhejiang 310006, China; 2 Department of Pharmacy, Second Affiliated Hospital, Zhejiang University School of Medicine, Hangzhou, Zhejiang 310009, China

**Keywords:** Coronary heart disease, Pharmacist, Secondary prevention, Mortality, Medication adherence

## Abstract

**Background:**

Secondary prevention is important for reducing both mortality and morbidity of patients with coronary heart disease (CHD). Pharmacists can provide medication and also work on disease management for patients with CHD. This review has been carried out to evaluate the role of pharmacist care on mortality, morbidity, and the CHD management.

**Methods:**

The PubMed, MEDLINE, EMBASE, Web of Science and Cochrane Central Register of Controlled Trials databases were searched for randomized controlled trials (RCTs) to evaluate the impact of pharmacist care interventions on patients with CHD (in both community and hospital settings). Primary outcomes of interest were mortality, cardiovascular events and hospitalizations. Secondary outcomes were medication adherence, blood pressure control, and lipid management.

**Results:**

Five RCTs (2568 patients) were identified. The outcomes were mortality, cardiovascular events, and hospitalizations in one study (421 patients), medication adherence in five studies, blood pressure in two studies (1914 patients), and lipid management in three studies (932 patients). The interventions of pharmacists included patient education, medication management, feedback to health care professionals, and disease management. There was no significant effect of pharmacist care on mortality, recurrent cardiac events or hospitalization of CHD patients. Significant positive effects of pharmacist care were shown on medication adherence in three studies, on blood pressure control in one study and on lipid management in one study.

**Conclusion:**

In this study, we concluded that pharmacists have a beneficial role in the care of CHD patients, although the evidence supporting positive impacts on mortality and morbidity remains uncertain due to the unavailability of data in these areas. Further research is needed to discern the contribution of pharmacist care on hard endpoints of CHD.

## Background

Coronary heart disease (CHD) is one of the leading causes of morbidity and mortality in the world [1]. With reference to increased survival rates after acute myocardial infarction and also due to an increase of the aging population, the burden of CHD increases gradually [1]. Secondary prevention is important because cardiovascular events occur at a high rate after an acute vascular event [2]. For example, about one fifth of patients were rehospitalized for ischemic heart disease or died within a year after the first acute coronary syndrome (ACS) [3].

Randomized studies have demonstrated the efficacy of lifestyle changes (e.g. smoking cessation, physical activity), and the use of medications such as aspirin, β-blockers, angiotensin-converting enzyme (ACE) inhibitors and statins to reduce death, reinfarction, or stroke in patients with CHD [4,5]. The nonadherence to medications for secondary prevention of CHD is associated with an increased risk of subsequent cardiovascular events and mortality [6-10]. Physicians and healthcare providers should make necessary efforts to engage the patient’s active participation in prescribed medical regimens and lifestyle changes to improve the prognosis of CHD.

Pharmacists, in addition to medication dispensing, can provide medication education and disease management for patients, to improve medication adherence to achieve the goals of desired therapeutic outcomes, and to improve safe medication use. Previous systematic reviews have demonstrated that interventions provided by pharmacists are beneficial in the management of major cardiovascular disease (CVD) risk factors in outpatients (e.g. lowering blood pressure and cholesterol levels or smoking cessation) [11], and in reducing the risk of hospitalizations in patients with heart failure [12]. The contributions from pharmacists in CHD secondary prevention have not been systematically reviewed so far, and hence we have carried out this study to evaluate the role of pharmacist care on mortality, morbidity, and the management of CHD.

## Methods

### Data sources and searches

A systematic literature search for randomized controlled trials (RCTs) on MEDLINE, PubMed EMBASE, Web of Science, and the Cochrane Central Register of Controlled Trials, from their inception until July 2012 was conducted (with an update performed in September 2013). Language restrictions were not applied. Search terms were pharmacy-related terms ('pharmacist’ OR 'pharmaceutical care’ OR 'pharmaceutical services’ OR 'pharmacy services’ OR 'hospital pharmacy’ OR 'community pharmacy’ OR 'pharmacy’) AND CHD-related terms ('coronary heart disease (CHD)’ OR 'coronary disease’ OR 'myocardial infarction (MI)’ OR 'angina pectoris’ OR 'revascularization’ OR 'coronary artery bypass grafting (CABG)’ OR 'percutaneous transluminal coronary angioplasty (PTCA)’ OR 'percutaneous coronary intervention (PCI)’ OR 'coronary artery stenting’) AND trial-related terms ('randomized controlled trial (RCT)’ OR 'clinical trial’ OR 'comparative study’). Additionally, the bibliographies of all relevant articles were reviewed.

### Study selection

**Figure 1:**
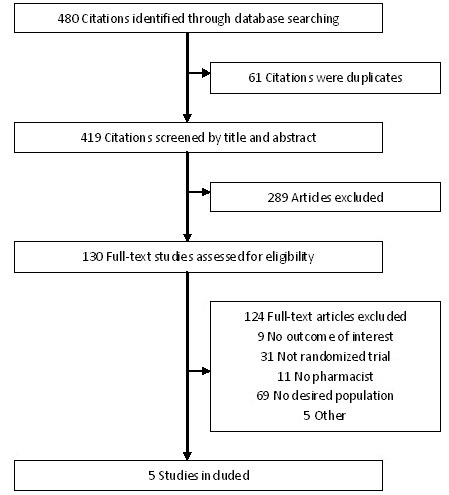
Flow diagram for identification, inclusion and exclusion of studies.

Two authors (HX and HC) independently screened the citations from the literature search to determine eligibility (Figure 1). Studies were included if they (1) had a randomized control design; (2) evaluated the impact of pharmacist care on patients with CHD (compared with usual care); and (3) had at least one of the outcomes of interest. Usual care for CHD involved routine care performed by a nurse, physician, and dispensing pharmacist. Pharmacist care in this study refers to enhanced pharmacist care provided by a clinical pharmacist, hospital pharmacist, community pharmacist, or pharmacy pharmacist. This study involves both pharmacist-directed care and pharmacist collaborative care. Primary outcomes of interest for this study were mortality, cardiovascular events and hospitalizations. Mortality included both cardiovascular and non-cardiovascular mortality. Cardiovascular events included non-fatal myocardial infarction, stroke, and coronary and carotid revascularization. Hospitalizations referred to the total number of cardiac-related or any-cause hospital admissions in the follow-up period. Secondary outcomes were medication adherence, blood pressure control, and lipid management.

Publications were excluded if they were not randomized, did not have adequate description of the pharmacist's intervention, did not directly apply to patients with CHD, were not conducted on patients all with CHD, or if they did not report the targeted outcomes. Disagreements were resolved by discussion.

### Data extraction and risk of bias assessment

Data extraction was independently performed by 2 authors (HX and YH) using a standardized data extraction form. Details about study design, participants, interventions, outcomes, risk of bias data and results were extracted. Risk of bias tools were applied as described in the Cochrane Handbook for Systematic Reviews of Intervention [13]. Factors that were considered included the following: the quality of random sequence generation and allocation concealment (selection bias), blinding of participants and personnel (performance bias), blinding of outcome assessment (detection bias), incomplete outcome data (attrition bias), selective reporting (reporting bias) and other bias (e.g. extreme baseline imbalance, fraudulence etc.). For each item, the quality characteristics of each study were rated as (1) low risk of bias; (2) unclear; and (3) high risk of bias.

## Results

Searches of the electronic databases identified 480 potential citations. After initial screening of titles and abstracts, 130 full-text studies were assessed for eligibility and five RCTs [14-18], all published in the English language, met the inclusion criteria. Figure 1 provides information on the number of studies identified, included and excluded, and the reasons for exclusion.

### Description of studies and types of interventions

Table 1 summarizes the characteristics of the included studies [14-18]. Overall, five studies involving a total of 2568 participants compared pharmacist interventions with usual care. Four studies were conducted in the United States (US) [14,16-18], and one in England [15]. The outcomes were mortality, cardiovascular events, and hospitalizations in one study (421 patients) [17], medication adherence in five studies [14-16,18], blood pressure in two studies (1914 patients) [15,17], and lipid management in three studies (932 patients) [16-18]. Three studies were conducted in hospital, outpatients clinics, or medical offices [16-18]; one study was conducted in community pharmacies [15]; and one in both hospital and community pharmacies [14]. One cluster RCT was randomized at clinic [18], and the remaining four trials were randomized at a patient level [14-17].

**Table 1:** Characteristics of included studies

**Source; country**	**Study setting**	**Study design, duration**	**Sample size (intervention/control)**	**Study participants; mean Age**	**Key components of pharmacist interventions**	**Intervention frequency**	**Description of usual care**	**Outcomes extracted**
Calvert [14], 2012; US	In hospital and community pharmacy	RCT, 6 months	143 (71/72)	CAD patients (UA or AMI; or ≥50% coronary occlusion on cardiac catheterization; or prior PTCA or CABG); 62 years	Focused medication counseling performed by the hospital study pharmacist, who identified and addressed barriers to medication adherence. A pocket medication card, a list of tips for remembering to take medications, and a pillbox were provided. Discharge medications were shared with the community pharmacist. The community pharmacist monitored for problems with adherence and communicated issues back to the patient and the patient’s care team	Every 6 weeks	Routine discharge counseling performed by the patient-care nurse and a letter/discharge summary from the hospital physician to the community physician	Medication adherence
The MEDMAN study [15], 2007; England	Community pharmacy	RCT, 12 months	1493 (980/513)	CHD patients (previous MI, angina, CABG and/or PTCA); 69 years	Consultations of therapy, medication compliance, lifestyle and social support were provided by the community pharmacist and recommendations were recorded and sent to the GP, who returned annotated copies to the pharmacists.	Depending on pharmacist-determined patient need	Usual care	Medication adherence and BP control
Faulkner [16], 2000; US	Outpatient clinic	RCT, 2 years	30 (15/15)	Patients 7 ~ 30 days after PTCA or CABG and baseline fasting LDL-C >130 mg/dl (3.3 mmol/L); 63 years	Pharmacist telephoned patients, emphasized on the importance of therapy, asked patients about when and where prescriptions were filled, how they paid for their prescriptions, potential side effects, overall well-being, and specific reasons for noncompliance when applicable.	Every week for 12 weeks	Counseling of appropriate use of the drugs and dietary instruction	Medication adherence and lipid management
Olson [17], 2009; US	Medical offices	RCT, 2 years	421 (214/207)	CAD patients (AMI, CABG, PCI) who had been enrolled in the CPCRS for at least 1 year and who had 2 consecutive controlled LDL-C, non–HDL-C, and blood pressure within 6 months before enrollment; 72 years	Review of laboratory results, blood pressure, medications and adherence, counseling on diet and exercise regimens, making medication adjustments, ordering follow-up laboratory tests, and mailing laboratory reminder letters for patients	Every 1 year	Usual care plus laboratory reminder letters	The occurrence of coronary events, mortality, and hospitalization; medication adherence, BP control, and lipid management
Straka [18], 2005; US	Outpatient clinic	cluster RCT, 6.5 months of active treatment, and 18 months of follow-up	481 (150/331)	CHD patients whose LDL-C levels were not at goal; 69 years	Managing lipid-lowering drug therapy and educating patients on cardiovascular risk reduction, communicating the responsible physician about the medication managements.	Every 6 weeks	Usual care	Medication adherence, BP control and lipid management

*Abbreviations*: *AMI* acute myocardial infarction, *BP* blood pressure, *CABG* coronary artery bypass graft, *CAD* coronary artery disease, *CHD* coronary heart disease, *CPCRS***C**linical **P**harmacy **C**ardiac **R**isk **S**ervice, *GP* general practitioner, *HDL-C* high-density lipoprotein cholesterol, *LDL-C* low-density lipoprotein cholesterol, *MEDMAN***Med**icines **Man**agement, *PCI* percutaneous coronary intervention, *PTCA* percutaneous transluminal coronary angioplasty, *RCT* randomized controlled trial, *UA* unstable angina, *US* United States.

The interventions delivered by pharmacists included (1) patient education (defined as education or counseling about therapy, medication compliance, lifestyle, social support etc.) in five studies [14-18]; (2) medication management (defined as medication review from medical records or patient interview; providing tools to improve medication compliance; assessment of medication compliance; monitoring of medication therapy such as assessment, adjustment, change of medications etc.) in five studies [14-18]; (3) feedback to health care professionals in three studies [14,15,18]; and (4) disease management (defined as assessment of targets for medication therapy such as blood pressure and lipid, and lifestyle such as smoking, obesity etc.) in four studies [15-18].

### Methodological quality of included studies

The studies were of variable methodological quality. Three studies provided evidence of adequate random sequence generation [14,16,17] and only two studies reported adequate concealment [14,15]. Because of the nature of the interventions, none of the studies blinded study participants to the pharmacist intervention, but two studies provided evidence of blinding assessment of outcome data [14,15]. High risks of bias existed in selection, performance and detection in the cluster RCT study [18].

### Primary outcomes

#### *Mortality, cardiac events, and hospitalizations*

Only one study reported all-cause mortality, the occurrence of fatal/nonfatal coronary events (acute MI, PCI, and CABG), and any-cause hospitalization as a secondary outcome of that study [17]. The study demonstrated that there was no difference in all-cause mortality, coronary events, or any-cause hospitalization between pharmacist care and the control group.

### Secondary outcomes

#### *Medication adherence*

All the five studies reported about medication adherence. Methods of medication adherence assessment and main outcomes of each study are shown in Table 2. Medication adherence was assessed by prescription in five studies [14,16-18], by patient self-reported in two studies [14,15], and by pill and package count in one study [16]. Adherence to aspirin and β-blocker were reported in two studies [14,15], lipid-lowering drug in five studies [14-18], and an ACE inhibitor in one study [15]. None of the studies demonstrated a statistically significant difference between pharmacist care and control in adherence to aspirin and an ACE inhibitor. Medication adherence was significantly increased in the intervention group than in the control group, for a β-blocker in one study [14], and for lipid-lowering drug in two studies [16,18].

**Table 2:** Summary of medication adherence measures in included studies

**Source**	**Method of measuring adherence**	**Medication involved**	**Outcome**
Calvert [14], 2012	Patient self-report and prescription records assessment	Aspirin, β-blocker, and lipid-lowering drug	No significant difference in self-reported adherence
			Better adherence to β-blocker in prescription assessed adherence in intervention than in control (*P* = 0.03)
The MEDMAN study [15], 2007	Patient self-report	Aspirin, lipid-lowering drug, β-blocker, and ACE inhibitor	No significant difference
Faulkner [16], 2000	Pill counts at 6 and 12 weeks and prescription records assessment at 1 and 2 years	Lipid-lowering drug	No significant difference at 6 or 12 weeks
			Medication compliance was significantly higher in intervention than in control (*P* < 0.05)
Olson [17], 2009	Prescription records assessment	Lipid-lowering drug	No significant difference
Straka [18], 2005	Prescription records assessment	Lipid-lowering drug	Medication compliance was higher in intervention than in control (78% versus 44.1%)

*Abbreviations*: *ACE* angiotensin-converting enzyme.

### Blood pressure (BP) control

Two studies reported the number of patients who achieved BP control target values [15,17]. One study demonstrated a statistically significant increase in BP control rate for pharmacist care compared with control [17] (Table 3).

**Table 3:** Summary of BP control and lipid management in included studies

**Source**	**Target for BP or lipid management**	**Outcome**
The MEDMAN study [15], 2007	BP: < 140/85 mmHg	No significant difference
Faulkner [16], 2000	LDL-C: ≤ 100 mg/dL (2.6 mmol/L)	No significant difference at 6 or 12 weeks
		More patients achieved target in intervention than in control at 1 and 2 years (*P* < 0.05)
Olson [17], 2009	LDL-C and non-HDL-C: < 100 mg/dL (2.6 mmol/L) and < 130 mg/dL (3.3 mmol/L) for all patients, < 70 mg/dL (1.8 mmol/L) and < 100 mg/dL (2.6 mmol/L) for patients with diabetes, multivessel coronary disease, at least 1 recurrent coronary event, or current smokers	No significant difference in maintaining LDL-C and non-HDL-C goal, and BP goal of < 130 mmHg
	BP: < 140/90 mmHg for all patients, <130/80 mmHg for patients with diabetes or CKD	More patients maintained a BP goal of < 140 mmHg in intervention than in control (*P* = 0.03)
Straka [18], 2005	LDL-C: ≤ 100 mg/dL (2.6 mmol/L)	More patients achieved LDL-C goal in intervention than in control at 6.5 months and the following 18 months (*P* < 0.001)

*Abbreviations*: *BP* blood pressure, *CKD* chronic kidney disease, *CVD* cardiovascular disease, *HDL-C* high-density lipoprotein cholesterol, *LDL-C* low-density lipoprotein cholesterol.

### Lipid management

Three studies reported the number of patients who achieved low-density lipoprotein cholesterol (LDL-C) control target levels [16-18]. Two studies demonstrated a statistically significant increase in LDL-C control rate for pharmacist care compared with control [16,18] (Table 3).

Two studies reported changes in lipid profiles in accordance with certain previous studies [16,18] and both of these studies reported a greater reduction in LDL-C levels with pharmacist care compared to controls.

## Discussion

Our systematic review, identified five RCTs (2568 patients) assessing the effects of pharmacist care in the secondary prevention of CHD. The 'dose’, 'duration’, method and outcome of pharmacist intervention varied across the studies. Our study did not show any survival benefits, or reduction in cardiac events and hospitalizations from pharmacist care in patients with CHD. However, it shows that the pharmacist can help to improve medication adherence, blood pressure and lipid control.

This review did not confirm the benefits of pharmacist intervention on mortality and morbidity of CHD. There are two possible explanations. First, as there are only a few trials available, with insufficient numbers of participants, there may not be adequate statistical power to detect clinical differences. Only one study used 'hard endpoints’ (such as mortality, cardiac events and hospitalizations) as secondary outcomes of the study, and the sample size was not estimated based on these [17]. Second, the design of this study was quite different from the others, where all of the patients received a disease management program from a clinical pharmacy specialist and had achieved target cholesterol values before randomization. The aim of this study was to evaluate whether patients with CHD discharged from the management program could maintain their lipid profile levels. Thus, even patients in the control group also received intensive pharmacist care before the study. Unfortunately, we could not find any other study comparing the pharmacist care to the 'real’ usual care for mortality, cardiac events and hospitalizations of patients with CHD. Further research is needed regarding the contribution of pharmacist care on mortality and morbidity of CHD.

Although there are no reports on the benefits of pharmacist intervention in mortality and morbidity of CHD, this review details the potential benefits of pharmacists on CHD care processes. Pharmacist care showed positive effects on medication adherence [14,16,18], blood pressure control [17] and lipid management [16,18]. Since medication nonadherence is associated with an increased rate of subsequent cardiovascular events and mortality [6-10], improvements in medication adherence will lead to clinically important reductions in recurrent myocardial infarctions and death.

## Conclusion

Due to the unavailability of data and the limited number of the studies, we could not carry out a quantitative meta-analysis. However, through qualitative analysis of the available data, we were able to evaluate the impact of pharmacist care on patients with CHD. However, the hypothesis that pharmacist care is beneficial for CHD care, with respect to mortality and morbidity, should be verified.

## Abbreviations

ACE: Angiotensin-converting enzyme; ACS: Acute coronary syndrome; BP: Blood pressure; CABG: Coronary artery bypass grafting; CHD: Coronary heart disease; CVD: Cardiovascular disease; LDL-C: Low-density lipoprotein cholesterol; MI: Myocardial infarction; PCI: Percutaneous coronary intervention; PTCA: Percutaneous transluminal coronary angioplasty; RCT: Randomized controlled trial.

## Competing interests

The authors declare that they have no competing interests.

## Author’s contributions

HX and HC designed the study, collected and analyzed the data, drafted the manuscripts, and are the guarantors of this study. HD and YH participated in the design of the study and collection of data. XY supervised the study and revised the manuscript. All authors approved the final manuscript.

## Pre-publication history

The pre-publication history for this paper can be accessed here:

http://www.biomedcentral.com/1472-6963/13/461/prepub
